# Cross-Theoretical Compliance: An Integrative Compliance Analysis of COVID-19 Mitigation Responses in Israel

**DOI:** 10.1177/00953997221140899

**Published:** 2022-12-22

**Authors:** Anne Leonore de Bruijn, Yuval Feldman, Christopher P. Reinders Folmer, Malouke E. Kuiper, Megan Brownlee, Emmeke Kooistra, Elke Olthuis, Adam Fine, Benjamin van Rooij

**Affiliations:** 1 University of Amsterdam, Netherlands; 2 Bar-Ilan University, Israel; 3 Arizona State University, USA; 4 University of California, Irvine, USA

**Keywords:** compliance, compliance theory, COVID-19, mitigation measures, social distancing, lockdown, stay-at-home measures, rational choice theory, deterrence, punishment, legitimacy, procedural justice, obligation to obey the law, social norms, capacity to comply, impulsivity, strain theory, negative emotions, opportunity to violate, trust in science, public health, Israel

## Abstract

To understand the question why people obey or break rules, different approaches have focused on different theories and subsets of variables. The present research develops a cross-theoretical approach that integrates these perspectives. We apply this in a survey of compliance with COVID-19 pandemic mitigation rules in Israel. The data reveal that compliance in this setting was shaped by a combination of variables originating from legitimacy, capacity, and opportunity theories (but not rational choice or social theories). This demonstrates the importance of moving beyond narrow theoretical perspectives of compliance, to a cross-theoretical understanding—in which different theoretical approaches are systematically integrated.

The COVID-19 pandemic has forced humanity to fundamentally change its behavior. During the initial wave of the pandemic, governments around the world adopted far-reaching restrictive measures, such as stay-at-home orders and social distancing rules, which were essential to slow the spread of the disease (Walker et al., 2020). The results have shown unprecedented behavioral change across most of the world. Compliance with these new measures has come with tremendous social and economic costs, as many people lost their jobs, education was severely disrupted, and ordinary life was completely upended. As such, the first pandemic wave created a unique and highly rare situation, where we see massive behavioral change induced by the adoption of governmental rules.

It is for this reason that compliance with mitigation measures during this period is of vital interest for the field of compliance studies. This field aims to understand the interaction between rules and human conduct (van Rooij & Sokol, 2021), and specifically, the way that compliance with rules, laws, and policies develops and is sustained. Compliance research has traditionally looked at these questions through particular theoretical perspectives, such as rational choice theories (Becker, 1968; Gul, 2009), social theories (Nolan & Wallen, 2021; Schultz et al., 2007), legitimacy theories (Tyler, 2006), capac- ity theories (Pratt & Lloyd, 2021; Van Rooij & Fine, 2021), and opportunity theories (Benson et al., 2009; Clarke, 2005; Felson, 1987). While these per- spectives have not been fully isolated, the focus in different strands of com- pliance literature has most often been to show how a particular theory can best explain compliance within a particular setting, or how one, two, or three theories interact (e.g., see Gneezy & Rustichini, 2000; Murphy, 2003; Thornton et al., 2005; Wenzel, 2005). As a result, the compliance literature exists as a patchwork of different theoretical strands, focusing on different settings and variables, which seldom have been combined in a systematic fashion. But although approaches that zoom in on a particular, limited subset of theories and variables may produce elegant results, their omission of concepts that are central in other strands of the literature may mean that important aspects of what shapes compliance are overlooked, limiting their capacity to explain or inform practical interventions. As such, the field of compliance studies faces an urgent challenge: it has not yet produced a perspective of compliance that integrates all main theoretical approaches into a systematic, cross-theoretical perspective, which understands compliance in relation to major compliance theories, identifies key variables as they apply to particular settings, and considers each to explore how compliance is shaped. The first pandemic wave provides a unique opportunity for developing and testing such a cross-theoretical perspective by providing a setting in which core mechanisms from all major compliance theories were operating, and their relationship with compliance can be studied.

The present research leverages this setting to address this major lacuna in the existing compliance literature. Our major purpose is to develop a cross- theoretical approach to compliance that bridges and integrates the major theoretical approaches in the field of compliance studies and to demonstrate how this can contribute beyond traditional, narrow approaches based on single theoretical perspectives. We develop this approach by studying compliance with COVID-19 mitigation measures during the first pandemic wave. For this purpose, we developed a cross-theoretical survey in which core variables from the major compliance theories were systematically assessed. We first examine how these variables relate to compliance when looked at from a single theoretical perspective. Then, we examine their associations with compliance when looked at from a cross-theoretical perspective in which the core variables from each theoretical perspective are considered simultaneously. Through this comparison, we demonstrate how in this setting, a cross-theoretical perspective can correct and enrich the insights obtained through narrower approaches to compliance. From this, we develop implications for our general understanding of compliance and the way that it might be studied in other settings.

We study these questions in the context of Israel. We do so firstly because this country adopted legally enforceable rules on social distancing and stay- at-home measures during the first pandemic wave, such that behavioral responses to these measures constitute compliance.^1^ Moreover, its response during this period leveraged core mechanisms from across the major compli- ance theories—including strong deterrence,^2^ social norms,^3^ measures to sus- tain citizens capacity to comply,^4^ and removal of opportunities for violating the rules.^5^ These features make Israel suitable for studying compliance from a cross-theoretical perspective. As with any setting, the selection of Israel, with its particular socio-cultural, economic, and legal dynamics, will limit the generalizability of the results obtained here. Accordingly, our aim is not to generalize empirically or to infer policy lessons for other jurisdictions or even to Israel itself in later periods. Rather, we will utilize the results on how single and cross-theoretical perspectives explain compliance in this setting to draw out broader theoretical lessons for compliance research and the way that this should be studied in other settings.

## Compliance Theories and Variables

The question why people obey or break rules is studied in different academic domains and for different types of rules. This spans subjects as diverse as tax evasion (in psychology and economics, e.g., Kirchler et al., 2008; Muehlbacher et al., 2011; Wenzel, 2005), street crime (in criminology, e.g., Weisburd, 2015), littering (in psychology, Keizer et al., 2008), environmental crime (e.g., Shover & Routhe, 2005; Yan et al., 2016), white-collar crime (in organizational science, e.g., Pusch & Holtfreter, 2021), and corruption (in anthropology, e.g., Haller & Shore, 2005). Different approaches have resulted in different theories of what shapes compliance. There are five main strands of theories: (1) rational choice theories, where people comply because the utility of compliance outweighs violation (Becker, 1968; Gul, 2009), (2) social theories, where people comply because they are influenced by opinions, values, and behaviors of others (Nolan & Wallen, 2021; Schultz et al., 2007), (3) legitimacy theories, where people comply out of a sense of duty that originates in the legitimacy of the legal system (Tyler, 2006), (4) capacity theories, where people comply because they are able to do so (Pratt & Lloyd, 2021; Van Rooij & Fine, 2021), and (5) opportunity theories, where people comply because they do not have the opportunity to violate the rules (Benson et al., 2009; Clarke, 2005; Felson, 1987).

### Rational choice theories

Rational choice approaches (Becker, 1968; Gul, 2009) hold that compliance originates from rational calculation of costs and benefits: people should comply if the benefits of compliance (minus its costs) are greater than the benefits of offending (minus its costs). A first major aspect of this is the cost of compliance (Donovan & Blake, 1992; Paternoster & Simpson, 1993). Complying with particular rules may lead people to suffer personal costs, such as inconvenience or expenses. Rational choice approaches would predict that as such personal costs increase, compliance will decrease. Conversely, compliance may also yield personal benefits, for example by helping to mitigate existing problems or threats (Donovan & Blake, 1992). As such perceived benefits increase, so should compliance.

Rational choice approaches to compliance have focused on punishment as an instrument that may critically alter the perceived costs and benefits of offending. General deterrence theory (Becker, 1968; Polinsky & Shavell, 2000; Shavell, 1991) holds that people will comply more frequently with rules when punishment is more certain and severe. Such perceptions have been found to be subjective, however (Apel, 2013; Decker et al., 1993); accordingly, it is especially people’s perceptions of punishment that are relevant (Grasmick & Bryjak, 1980). Thus, people should comply more when they see punishment for not complying as more certain and severe.

### Social theories

Social theories focus on the social embeddedness of people’s conduct. This dimension is prominent, for example, in social norms theories of compliance in psychology (Nolan & Wallen, 2021; Nolan et al., 2008; Schultz et al., 2007), social learning and subcultural theories in criminology (Akers & Jensen, 2011; Pratt et al., 2010; Wolfgang et al., 1967), and code of the street theory and normalization of deviancy ideas in sociology (Anderson, 2000; Vaughan, 1997). Such theories hold that people’s responses to the law are deeply embedded in a social context, which may (consciously or unconsciously) shape their decisions to comply. Accordingly, the more that people see others break legal rules, the more likely they should be to do so them- selves (Cialdini & Trost, 1998; Cialdini et al., 2006; Goldstein et al., 2008; Schultz et al., 2007).

### Legitimacy theories

Legitimacy theories generally hold that compliance with rules or authorities derives from their perceived legitimacy. Legitimacy here is “a property or quality of possessing rightful power and the subsequent acceptance of, and willing deference to, authority” (Jackson & Gau, 2016). The more that people see rules or authorities as legitimate, the more they should comply with them.

Such acceptance and deference will first of all be greater when people agree substantively with the authorities on the rules that they adopt. The greater this moral alignment is, the greater should be their compliance (Jackson et al., 2012; Tyler, 2006). Legitimacy is also higher when people see authorities and rules as proper and just; accordingly, people also should comply more when they have a favorable view of the authorities (Jackson et al., 2012, 2013). Closely related to this are perceptions of their procedural fairness: how fair their decision-making procedures and one’s treatment by them are seen to be (Tyler, 2006). The more that people regard rules and their implementation as procedurally fair, the more that they should comply with them (Walters & Bolger, 2019).

### Capacity theories

Capacity theories hold that people’s compliance with rules depends on their capacity to comply. They reflect the notion that people’s personal or practical circumstances may make it easier or more difficult for them to do what rules request of them. For example, people may lack the knowledge, resources, or the discretion that is necessary in order for them to comply (Gray & Silbey, 2011; Winter & May, 2001; Yan et al., 2016). This idea is also reflected in criminological research that suggests that people’s compliance with rules may depend on their ability to exert self-control (Gott- fredson & Hirschi, 1990; Hirschi & Gottfredson, 1993; Pratt & Cullen, 2000; Pratt & Lloyd, 2021), or on strenuous, negative emotional states that people may cope with through rule breaking (Agnew, 2007; Piquero & Sealock, 2004). As such, people should comply more the greater their practical capac- ity to do so is, the greater their self-control, and the less that they experience negative emotions.

### Opportunity theories

Opportunity theories link compliance to opportunities for breaking rules derived from the situation at hand (Benson et al., 2009; Feldman, 2018; Van Rooij & Fine, 2021). Routine activities theory holds that criminal behavior develops more easily when there are attractive targets that are left undefended to motivated offenders (Cohen & Felson, 1979; Osgood et al., 1996; Spano & Freilich, 2009). Situational crime prevention theory has broadened this idea toward all situations that make illegal behavior easier, for instance by providing easy access to tools or techniques needed to break the law (Clarke, 2003, 2005). According to this logic, people should comply more the less opportunity they have (or perceive they have) for breaking particular rules.

## Integration of Theoretical Strands in Existing Compliance Literature

These major theoretical strands in the compliance literature have developed in relative isolation from each other, in different fields, and focused on different behaviors and settings. Within each of these domains, exten sive work has examined how a particular theory, or combination of theories, can best explain compliance within a particular setting. Studies that analyze compliance through the lens of multiple theories have mostly focused on combinations of two or three theories, and draw from these theories only a limited subset of variables (e.g., see Gneezy & Rustichini, 2000; Murphy, 2003; Thornton et al., 2005; Wenzel, 2005). Some studies have brought together variables from across a larger number of theoretical strands (e.g., Nielsen & Parker, 2012; Winter & May, 2001; Yan et al., 2016); yet even these have not sought to do so systematically. For instance, such studies overlook opportunities for offending, and they fail to fully or properly incorporate capacity theories; Nielsen and Parker (2012), for example, do not incorporate capacity at all; Winter and May (2001) measure people’s confidence in their legal knowledge, rather than their actual legal knowledge; and Yan et al. (2016) focus on financial capacity and legal knowledge, but without looking systematically at other core capacities their participants (Chinese farmers) may need to comply with pesticide rules.

The reasons for the narrow focus of existing compliance research are manifold. To begin with, different literatures have developed in parallel, in relative isolation from each other (van Rooij & Sokol, 2021). Furthermore, the different disciplines that study these questions have different research traditions that lead them to focus on different levels and units of analysis (Becher, 1994; Biglan, 1973; Matsueda, 2017). Moreover, the social sciences tend to face incentives that favor simple models over more complex ones (Baker, 2016; Bernstein et al., 2000; Mearsheimer & Walt, 2013; Swedberg, 2012). As such, the narrow focus of existing compliance research may also reflect an academic preference for developing neat statistical models that utilize a small subset of variables, where clear hypotheses about main effects and interactions can be formulated and tested. Although such studies have amassed considerable evidence on how focal variables may shape compliance in particular settings, important questions can be raised over such narrow explanations. In particular, the notion that they omit key theories and variables that are central in other segments of the literature suggests that these perspectives may overlook important facets of what drives compliant behavior. Indeed, it is possible that variables that are influential in such narrow approaches might show different associations with compliance once a wider spectrum of relevant variables is considered (e.g., omitted variable bias, Wilms et al., 2021). This risk could have severe consequences by undermining not only the robustness of such scientific conclusions, but also the value of practical initiatives that seek to promote compliance by building on insights from the research. There is an urgent need for a cross-theoretical perspective that integrates the major compliance theories and examines systematically how their core variables jointly come to shape compliance within a particular setting.

## Cross-Theoretical Perspective on Compliance

In sum, compliance models may have omitted variable bias, and they may not reflect how compliance happens in practice or in the contexts that shape compliance behavior. For example, one setting might emphasize social components of compliance, while other settings might emphasize the benefits of compliance, appealing to an individual’s rationality. Still, some settings may use a combination of both or incorporate various elements of compliance models to encourage compliance. The present study develops a cross-theoretical approach that bridges and integrates the five major theoretical approaches in the compliance literature: rational choice, social, theories, legitimacy, capacity, and opportunity theories. Our approach proposes that to understand compliance within a particular setting, core variables from each of these theoretical strands should be considered simultaneously and systematically. Moreover, we argue that such a cross-theoretical approach can reveal fundamentally different conclusions about what shapes compliance, which may be obscured in narrow approaches based on single theoretical perspectives. This paper uses the case of Israel’s response to the COVID-19 pandemic as a case of multifaceted compliance, demonstrating the omitted variable problem of conventional models and the need for a cross theoretical approach.

## Mitigation Measures in Israel

Israel’s response to COVID-19 during the first pandemic wave was gradual. Beginning on March 11, 2020, social distancing measures were imposed, and large group gatherings (with over 100 people) were banned.^6^ On March 19, the government declared a national state of emergency, and strict stay-at-home measures were adopted.^7^ These ordered citizens to not leave their homes except for essential activities, banned all public gatherings, apart from some religious and family activities (e.g., small-scale funerals and circumcisions), and closed almost all retail shops and restaurants, except for deliveries (for more details, see Last, 2020; Waitzberg et al., 2020). These measures were further tightened on March 25.^8^ According to the University of Oxford’s COVID-19 Government Response Tracker,^9^ the Israeli measures during this period were among the strictest in the world, with a score of 90.74. In order to promote compliance with these measures, Israel developed a range of interventions, which leveraged several of the core mechanisms from across the major compliance theories. To begin with, the government sought to convince citizens of the benefit of complying by focusing on the costs of violation, through deterrence based on punishment. Violation of the measures was a criminal offense, punishable by fines of up to 5,000 shekels ($1,522) and up to six months of imprisonment.^10^ By April 12, authorities had fined 25,837 citizens and businesses for violating the mitigation measures, especially for being more than 100 meters away from their place of residence. Furthermore, usage of technological surveillance was widespread, including phone tracking by the Israel Security Agency^11^ and the deployment of the military to back up regular law enforcement and to gather large-scale data.^12^ Additionally, Israeli authorities adopted a range of financial measures to compensate citizens for the costs associated with COVID-19 mitigation measures, such as loss of income and employment.^13^

Second, authorities sought to convince citizens of the benefits of complying, for example by emphasizing the threat of the disease, and the role of mitigation measures to counter this. Prime Minister Netanyahu stated: “What it takes for each and every one of you, beyond complying with official guidelines, is strict self-discipline. If you don’t enlist to protect yourself and your families, this will be a disaster.” He added, “You have to stay home, stay in the houses - stay alive, the danger lurks for each of you.”^14^ Similarly, Health Ministry Director-General Moshe Bar Sim-Tov’s remarked: “The scenarios that happen in Italy and Spain can happen here as well. Our ability to really avoid such extreme scenarios depends on your behavior.”^15^

Third, the authorities sought to instill social norms for complying. For example, the Ministry of Health and the Local Government Center collabo- rated with a private company to create an index of compliance with social distancing measures for Israel’s biggest 76 cities (active until May 2, 2020).^16^ By doing so, it sought to enable local authorities to make salient the local norms for compliance and thereby inspire others to comply (for more infor- mation, see Barak et al., 2021).

Fourth, authorities took measures to ensure that communities had the capacity to comply. For example, the Israeli military provided food to ultra- orthodox communities under lockdown, and offered help to place patients from these communities in hotels if they could not self-isolate at home.^17^ Furthermore, during the Passover holiday, all non-essential government and local authority workers were placed on paid leave, enabling them to stay at home without losing income.^18^

Last, authorities sought to reduce opportunities for violating. They did so initially by prohibiting large-scale gatherings,^19^ and closing schools and universities.^20^ Eventually all public gatherings were banned (apart from some small-scale religious and family activities), and nearly all stores, restaurants and other public places were closed (see Waitzberg et al., 2020). By doing so, the authorities removed important opportunities for citizens to leave the house and get close to others. In a similar vein, the authorities in some instances implemented closures of particular neighborhoods^21^ or even cities with high rates of infections,^22^ in order to limit opportunities for contact between residents and outsiders.

## Present Study

We operationalized our cross-theoretical approach by developing a survey, which measured core variables from each of the five major theoretical approaches in the compliance literature. This instrument was previously used to study compliance with COVID-19 mitigation measures in the United States (Van Rooij et al., 2020), the United Kingdom (Kooistra et al., 2020), and the Netherlands (Kuiper et al., 2022). The survey assessed participants’ perceptions of the costs of complying with mitigation measures, the threat of the virus, the certainty of punishment, and the severity of punishment (drawn from rational choice theories); social norms for complying with mitigation measures (social theories); moral alignment with mitigation measures, authority evaluation, felt obligation to obey the authorities, and procedural justice (legitimacy theories); capacity to comply, impulsivity, and negative emotions (capacity theories); and perceived opportunities for violating mitigation measures (opportunity theories). These measures were derived from validated instruments and previous research (for more information, see the Supplemental Appendix).

To assess compliance, the survey looked at two core contagion mitigation measures imposed during the first pandemic wave: social distancing and stay-at-home measures. First, the survey looks at whether people kept a safe distance from others (in Israel this has been defined at 2 meters)^23^ and whether they refrained from meeting people outside of their own household. Second, stay-at-home-type measures required people to stay at home and go out only for what are deemed essential activities (defined in the Israeli context as anything related to medial needs, food purchasing, and jobs that have been defined as essential).^24^ To study compliance with these measures, the survey used self-report measures which asked respondents to report the frequency with which they have complied with these measures.

As noted, the major purpose of our research was to develop a cross-theoretical approach to compliance, and to demonstrate how this contributes beyond traditional, narrow approaches based on single theoretical perspectives. We do so by analyzing compliance with pandemic mitigation measures during the first pandemic wave in Israel. The intended contribution of our research is theoretical and methodological: to demonstrate how understanding compliance requires the integration of the major theoretical strands in the compliance literature. In light of this, our research did not aim to evaluate the policy response in Israel or to zoom in on the country’s particular social, religious, and political dynamics (see, e.g., Barak Corren & Perry-Hazan, 2021; Gesser-Edelsburg et al., 2020; Waitzberg et al., 2020). For this reason, we did not seek to collect a sample that was fully representative of the Israeli population.

## Method

### Participants

A total of 509 Israeli residents completed the survey in Qualtrics. Participants were recruited through Panel4All, an online survey company in Israel. For this study, a representative Hebrew-speaking sample was drawn. They were recruited using a stratified sampling approach, in which the final intended sample size was divided into subgroups with the same demographic proportions (age, gender, religiosity, and residency) as the Hebrew-speaking population based on estimates from the Israel Central Bureau of Statistics (https://www.cbs.gov.il/en). The data were collected from April 7 to 9th, 2020 and can be accessed through links in the Appendix. Participants were paid approximately six NIS for their participation.^25^ Eleven participants were excluded because they provided professional care for COVID-19 patients, and 94 others were excluded because they failed the attention check.^26^ Relative to the general Israeli population, the final sample was older, more female, and more Jewish, and included fewer Orthodox and Ultra-Orthodox participants. Table 1 shows the characteristics of the final sample (*N*=411).

**Table 1.** Descriptive Statistics of Demographic and Control Variables (*N* = 411).

	Mean (*SD*)	%
Age	40.36 (15.01)	
Gender		
Male		47.9
Female		52.1
Ethnicity		
Jewish		84.4
Arabic		14.4
Other		1.2
Religion		
Secular		40.6
Traditional		29.0
Orthodox		8.8
Ultra-orthodox		6.1
SES pre-COVID-19	4.93 (1.62)	
SES post-COVID-19	5.26 (1.93)	
Education		
Some high school, no diploma		9.2
High school graduate, diploma		23.1
Some college credit, no degree		6.8
Associate degree		14.6
Bachelor’s degree		30.2
Master’s degree		14.8
Professional degree		0.2
Doctorate degree		1.0
*N* people in household	4.39 (1.49)	
*N* children	1.79 (1.21)	
Friends/family 75+		49.1
Health issues self		19.2
Health issues others		71.3
Trust in science	4.10 (0.92)	
Trust in media	3.26 (1.19)	
Political orientation		
Very progressive		25.1
Slightly progressive		26.3
Slightly conservative		21.9
Very conservative		8.8

*Note*. For political orientation and religion, percentages may not add up to 100% as respondents could indicate that they preferred to not answer these questions.

### Materials

A detailed description of the materials, including a complete list of items for every scale, is displayed in the Supplemental Appendix. Here, we provide an overview and briefly describe the variables.

#### Demographic and control variables

The following demographic and control variables were recorded: age, gender, ethnicity, religion (secular, traditional, orthodox, or ultra-orthodox),^27^ education, number of people in household, number of children, and social economic status before and after COVID-19 (based on the MacArthur Scale of Subjective Social Status; Adler et al., 2000). Furthermore, we measured whether participants provided professional care for COVID-19 patients, they had friends or family over the age of 75, and whether they or anyone they knew had underlying health issues that placed them at increased risk of COVID-19. Last, we measured participants’ trust in science and trust in media (adapted from McCright et al., 2013) and their political orientation (adapted from Fine et al., 2019; Hasson et al., 2018; Wojcik et al., 2015).^28^ Descriptive statistics for these variables are shown in Table 1.

#### Compliance with COVID-19 measures

Our compliance measures did not display sufficient internal consistency to be combined into a scale measure (α= .64). For this reason, we decided instead to analyze not the *degree* of compliance (as captured by Likert measures), but rather the *frequency* of compliance: that is, the frequency that participants reported that they (fully) complied with the mitigation measures (or did not). Consider that the behavioral rules prescribe that people should keep a safe distance from others outside of their household, and that they should stay at home except for essential activities. This means that only people who reported that they had always done so (7 = “always”) had fully complied with these rules. Conversely, people who indicate another response option (6 = “almost *always*,” 5 = “usually,” 4 = “frequently,” 3 = “occasionally,” 2 = “almost *never*,” 1 = “never”) thus report that they (sporadically or continually) did not comply with these measures. As such, an alternative way of analyzing these data is to count the number of instances that participants reported full compliance (7 = “always”). The approach of analyzing offenses as count variables, and analyzing these with Poisson regression, is commonly accepted as a sound (and indeed preferred) method of analyzing rule violating behavior in other fields that study compliance, such as criminology (e.g., see MacDonald & Lattimore, 2010; Trinkner et al., 2018).

To do so, we firstly recoded each individual (Likert) item into a binary measure of full compliance (1=complied fully; 0=did not comply fully). Next, we counted the frequency of full compliance across the five items, yielding a count variable that varied from 0 (non-compliant on all five items) to 5 (compliant on all five items). We utilized this measure as the dependent variable in our analyses.

#### Rational-choice theories

Four variables rooted in rational choice approaches to compliance were assessed: (1) perceived costs of compliance (α = .77), (2) threat perceptions (*r* = .56, *p* < .001), (3) perceived punishment certainty (α = .87), and (4) perceived punishment severity (*r* = .88, *p* < .001).

#### Social theories

One variable rooted in social approaches to compliance was measured: perceived descriptive social norms (*r* = .94, *p* < .001).

#### Legitimacy theories

Four variables reflecting legitimacy and procedural jus- tice were assessed: (1) moral alignment (*r* = .86, *p* < .001), (2) authority evaluation (α = .86), (3) obligation to obey (including general obligation to obey the law (OOL) and non-normative obligation to obey), and (4) procedural justice (α = .95).

#### Capacity theories

Three variables derived from capacity approaches to com- pliance were measured: (1) capacity to comply (α = .81), (2) impulsivity (α = .75), and (3) negative emotions (α = .87).^29^

#### Opportunity theories

One variable rooted in opportunity approaches to com- pliance was assessed: opportunity to violate (α = .86).

## Results

### Analysis Strategy

To understand how the different compliance mechanisms relate to compli- ance when looked at from a single theoretical perspective, we firstly exam- ine correlations between each mechanism and compliance. Next, we conduct (Poisson) regression analysis to understand what shapes compliance when looked at from an integrative, cross-theoretical perspective.

### Descriptive Statistics

Table 2 shows the descriptive statistics of the compliance measures. For all items, means were large, indicating that overall participants reported high rates of compliance. Table 3 shows the descriptive statistics of the independent variables.

**Table 2.** Descriptive Statistics of Compliance Measures.

Item	Mean (*SD*)	Binary (% full compliance)
Social distancing measures		
I still meet people outside of my direct household^a^	5.69 (1.53)	35.0
I keep a safe distance from people outside of my direct household	5.92 (1.48)	47.7
I still visit others (friends, relatives) outside of my direct household^a^	6.48 (1.04)	67.9
I still allow others (friends, relative) to visit my direct household^a^	6.47 (1.06)	68.9
Stay-at-home measures		
I have stayed at home after I was ordered to do so, apart from engaging in essential activities (e.g., grocery shopping, medical appointments)	6.22 (1.06)	60.3
Average frequency of full compliance (count variable)	2.80 (1.67)	

a Reverse coded.

**Table 3.** Descriptive Statistics of Independent Variables.

Variables	*M* (*SD*)	Scale
Rational choice theories		
Costs of compliance	3.52 (1.45)	1–7
Perceived threat	5.21 (1.38)	1–7
Punishment certainty	4.28 (1.67)	1–7
Punishment severity	3.48 (1.43)	1–6
Social theories		
Descriptive social norms	5.44 (1.34)	1–7
Legitimacy theories		
Moral alignment	6.39 (0.98)	1–7
Authority evaluation	5.04 (1.21)	1–7
General OOL	5.70 (1.37)	1–7
Non-normative obligation	5.22 (1.38)	1–7
Procedural justice	5.85 (1.06)	1–7
Capacity theories		
Practical capacity to comply	5.99 (1.24)	1–7
Impulsivity	1.97 (0.81)	1–5
Negative emotions	3.98 (1.33)	1–7
Opportunity theories		
Opportunity to violate	2.44 (1.37)	1–7

### Correlations

We first explored the correlations between compliance and the demographic and control variables to identify relevant covariates (Table 4). Four variables showed significant correlations with compliance: ethnicity, number of children, health issues placing oneself at increased risk, and trust in science. As such, these variables were used as covariates in the main regression analysis.

**Table 4.** Kendall’s Tau Correlations of Control Variables (*N* = 411).

	1	2	3	4	5	6	7	8	9	10	11	12	13	14	15	16
1. Age	—															
2. Gender	−.023	—														
3. Ethnicity	−.175^b^	.033	—													
4. Religion	.030	.007	n/a^30^	—												
5. Education	.006	−.084	.114^a^	−.105^a^	—											
6. *N* Household	.007	−.066	.072	.244^b^	−.079^a^	—										
7. *N* Children	.038	.012	.061	.149^b^	.076	.424^b^	—									
8. SES pre-COVID-19	−.020	.115^b^	−.022	−.003	−.099^a^	−.036	−.048	—								
9. SES post-COVID-19	−.051	.164^b^	.060	.002	−.105^b^	−.018	.050	.591^b^	—							
10. Friends/family 75+	−.001	−.060	.031	−.061	.131^b^	−.027	.083	−.148^b^	−.078	—						
11. Health issues self	.040	−.088	−.097	−.001	.085	−.167^b^	−.091^a^	.061	.087^a^	.027	—					
12. Health issues other	.007	.155^b^	−.044	−.052	.094^a^	−.056	.041	−.017	.032	.204^b^	.187^b^	—				
13. Trust in science	.048	−.136^b^	−.033	−.118^a^	.082^a^	−.076	−.017	−.092^a^	−.111^b^	.040	.059	.088	—			
14. Trust in media	.054	.056	−.001	−.168^b^	−.019	−.028	−.017	−.023	−.046	.051	.020	.062	.303^b^	—		
15. Political orientation	.049	−.123^a^	−.129^a^	.430^b^	−.058	.121^b^	.053	−.063	−.065	−.068	.016	−.043	−.093^a^	−.205^b^	—	
16. Compliance	.037	.083	−.120^b^	.046	−.046	−.023	−.088^a^	.046	.031	−.072	.102^a^	.044	.091^a^	.035	−.019	—

*Note.* Ethnicity: *N* = 406; religion: *N* = 347; political orientation: *N* = 337.

a Correlation is significant at the .05 level.

b Correlation is significant at the .01 level.

Next, we examined the correlations between compliance and the independent variables (Table 5). There were significant correlations between compliance and mechanisms from all five of the major families of compliance theories: perceived threat and punishment certainty (rational choice theories), social norms (social theories), moral alignment, authority evaluation, general obligation to obey the law, non-normative obligation, and procedural justice (legitimacy theories), practical capacity to comply and impulsivity (capacity theories), and opportunity to violate (opportunity theories). These variables were therefore entered as predictors in the main regression analyses. Conversely, three variables did not yield statistically significant correlations with compliance: costs of compliance and punishment severity (rational choice theories) and negative emotions (capacity theories). These variables were not included in the main analyses.

**Table 5.** Kendall’s Tau Correlations of Independent Variables (*N* = 411).

	1	2	3	4	5	6	7	8	9	10	11	12	13	14	15
1. Costs of compliance	—														
2. Perceived threat	.013	—													
3. Punishment certainty	.096^b^	.078^a^	—												
4. Punishment severity	−.105^b^	−.065	−.109^b^	—											
5. Social norms	−.058	−.002	.175^b^	−.037	—										
6. Moral alignment	−.061	.340^b^	.128^b^	.001	.201^b^	—									
7. Authority evaluation	−.148^b^	.157^b^	.016	−.043	.120^b^	.260^b^	—								
8. General OOL	−.062	.170^b^	.086^a^	.031	.201^b^	.348^b^	.166^b^	—							
9. Non-normative obligation	.085^a^	−.080^a^	.043	−.107^b^	−.119^b^	−.221^b^	−.065	−.155^b^	—						
10. Procedural justice	−.067	.018	.053	.011	.193^b^	.114^b^	.020	.077^a^	−.151^b^	—					
11. Practical capacity to comply	.016	.073	.150^b^	.037	.292^b^	.350^b^	.090^a^	.230^b^	−.168^b^	.201^b^	—				
12. Impulsivity	.140^b^	−.047	−.053	−.049	−.121^b^	−.180^b^	−.040	−.186^b^	.182^b^	−.111^b^	−.134^b^	—			
13. Negative emotions	.169^b^	.159^b^	.090^b^	−.125^b^	−.024	.012	−.007	−.034	.174^b^	−.074^a^	.027	.171^b^	—		
14. Opportunity to violate	−.009	−.119^b^	−.077^a^	−.038	−.182^b^	−.286^b^	−.109^b^	−.271^b^	.145^b^	−.047	−.325^b^	.165^b^	.018	—	
15. Compliance	−.028	.153^b^	.148^b^	.050	.270^b^	.370^b^	.087^a^	.275^b^	−.149^b^	.140^b^	.475^b^	−.151^b^	.020	−.373^b^	—

a Correlation is significant at the .05 level.

b Correlation is significant at the .01 level.

### Regression Analyses

As our dependent variable constitutes a non-categorical count variable that assumes discrete values (i.e., the number of reported instances of full compliance: 0, 1, 2, 3, 4, or 5), we relied on multivariate Poisson regression, which is suitable for such models (Coxe et al., 2009; Maddala, 1986). To under- stand how the different predictors each related to compliance when looked at from a single theoretical perspective, we first estimated 11 separate regres- sion models: one for each of the independent variables identified in the previ- ous analysis (respectively perceived threat, punishment certainty, social norms, moral alignment, authority evaluation, general obligation to obey the law, non-normative obligation, procedural justice, practical capacity to comply, impulsivity, and opportunity to violate). The previously identified demographic and control variables were included as covariates (i.e., ethnicity, number of children, health issues placing oneself at increased risk, and trust in science).

Table 6 reports the results of these analyses. When looked at from a single theoretical perspective, perceived threat and punishment certainty (rational choice theories), social norms (social theories), moral alignment, general obligation to obey the law, and non-normative obligation (legitimacy theo- ries), practical capacity to comply and impulsivity (capacity theories), and opportunity to violate (opportunity theories) all were significant predictors of compliance. Indeed, for every one-unit increase (holding constant all other variables in the model), compliance would be expected to increase by factors of 1.07 (95% CI 1.02–1.12, *p* = .003) for perceived threat; of 1.07 (95% CI 1.03–1.11, *p* < .001) for punishment certainty; of 1.11 (95% CI 1.06–1.17, *p* < .001) for social norms; of 1.33 (95% CI 1.23–1.44, *p* < .001) for moral alignment; of 1.16 (95% CI 1.11–1.22, *p* < .001) for general obligation to obey the law; and of 1.33 (95% CI 1.25–1.42, *p* < .001) for practical capacity to comply. Conversely, for every one-unit increase (holding constant all other variables in the model), compliance would be expected to decrease by factors of .95 (95% CI 0.92–0.98, *p* = .005) for non-normative obligation; of .89 (95% CI 0.83–0.97, *p* = .005) for impulsivity; and of .83 (95% CI 0.79–0.87, *p* < .001) for opportunity to violate. Authority evaluation and procedural justice (legitimacy theories) did not have significant effects on compliance in these analyses. In sum, when looked at from a single theoretical perspective, focusing on a narrow subset of predictors, all five theoretical families appear to contribute significant predictors of compliance.

**Table 6.** Poisson Regression Models.

	Model 1	Model 2	Model 3	Model 4	Model 5	Model 6	Model 7	Model 8	Model 9	Model 10	Model 11
Intercept	.41^a^ (.20)	.45^a^ (.19)	.20 (.21)	−.96^b^ (.30)	.54^b^ (.20)	−.00 (.21)	.92^c^ (.19)	.45^a^ (.23)	−.96^c^ (.26)	.99^c^ (.20)	1.18^c^ (.18)
Control variables											
Ethnicity (Arab)	−.23^a^ (.09)	−.28^b^ (.09)	−.18 (.09)	−.20^a^ (.09)	−.23^a^ (.09)	−.24^a^ (.09)	−.25^b^ (.09)	−.23^a^ (.09)	−.21^a^ (.09)	−.19^a^ (.10)	−.28^b^ (.09)
*N* children	−.05 (.03)	−.05 (.03)	−.05 (.03)	−.05^a^ (.03)	−.05 (.03)	−.05^a^ (.03)	−.05 (.03)	−.05 (.03)	−.03 (.03)	−.05 (.03)	−.05 (.03)
Health issues self	.09 (.07)	.09 (.07)	.11 (.07)	.10 (.07)	.12 (.07)	.09 (.07)	.10 (.07)	.10 (.07)	.09 (.07)	.10 (.07)	.09 (.07)
Trust in science	.07 (.03)	.07^a^ (.03)	.05 (.03)	.03 (.03)	.07^a^ (.03)	.04 (.03)	.06 (.03)	.07^a^ (.03)	.05 (.03)	.06 (.03)	.06 (.03)
Rational choice theories											
Perceived threat	.07^b^ (.02)										
Punishment certainty		.07^c^ (.02)									
Social theories											
Social norms			.11^c^ (.02)								
Legitimacy theories											
Moral alignment				.29^c^ (.04)							
Authority evaluation					.03 (.02)						
General OOL						.15^c^ (.02)					
Non-normative obligation							−.05^b^ (.02)				
Procedural justice								.05 (.03)			
Capacity theories											
Capacity to comply									.29^c^ (.03)		
Impulsivity										−.11^b^ (.04)	
Opportunity theories											
Opportunity to violate											−.19^c^ (.02)

a *p* < .05. ^b^*p* < .01. ^c^*p* < .001.

Next, we estimated a regression model in which variables from all theo- retical approaches were considered simultaneously. In this cross-theoretical model, all 11 predictors (perceived threat, punishment certainty, social norms, moral alignment, authority evaluation, general obligation to obey the law, non-normative obligation, procedural justice, practical capacity to comply, impulsivity, and opportunity to violate) were entered simultaneously. The model again controlled for the previously identified demographic and control variables. Table 7 displays the results.

**Table 7.** Poisson Regression Multivariable Model.

Predictor	*B*	*SE*	95% Confidence interval		Test statistic	*p* Value	Exp (*B*)
			Lower	Upper			
Intercept	−1.18	0.42	−1.99	−0.36	−2.82	.005^b^	.31
Control variables							
Ethnicity (Arab)	−.24	0.10	−0.44	−0.04	−2.42	.016^a^	.79
*N* children	−.04	0.03	−0.09	0.01	−1.61	.108	.96
Health issues self	.06	0.07	−0.08	0.20	.80	.421	1.06
Trust in science	.02	0.03	−0.05	0.09	.60	.551	1.02
Rational choice theories							
Perceived threat	.02	0.02	−0.03	0.07	.85	.393	1.02
Punishment certainty	.03	0.02	−0.01	0.06	1.51	.132	1.03
Social theories							
Social norms	.02	0.02	−0.03	0.06	.66	.509	1.02
Legitimacy theories							
Moral alignment	.11	0.05	0.02	0.20	2.37	.018^a^	1.12
Authority evaluation	−.02	0.02	−0.06	0.02	−1.02	.308	.98
General OOL	.06	0.03	0.01	0.11	2.34	.019^a^	1.06
Non-normative obligation	−.01	0.02	−0.05	0.03	−.57	.566	.99
Procedural justice	−.02	0.03	−0.08	0.04	−.64	.522	.98
Capacity theories							
Capacity to comply	.19	0.03	0.13	0.26	5.64	.000^c^	1.21
Impulsivity	.01	0.04	−0.07	0.09	.31	.755	1.01
Opportunity theories							
Opportunity to violate	−.09	0.02	−0.14	−0.04	−3.76	.000^c^	.91

a *p* < .05. ^b^*p* < .01. ^c^*p* < .001.

The results revealed four variables that were statistically significant predictors of compliance: moral alignment and general obligation to obey the law (legitimacy theories), capacity to comply (capacity theories), and opportunity to violate (opportunity theories). For every one-unit increase (holding constant all other variables in the model), compliance would be expected to increase by factors of 1.12 (95% CI 1.02–1.22, *p* = .018) for moral alignment; of 1.06 (95% CI 1.01–1.12, *p* = .019) for general obligation to obey the law; and of 1.21 (95% CI 1.13–1.30, *p* = .000) for practical capacity to comply. Conversely, for every one-unit increase (holding constant all other variables in the model), compliance would be expected to decrease by a factor of .91 (95% CI 0.87–0.96, *p* = .000) for opportunity to violate.

Conversely, other variables, which had shown significant associations with compliance when looked at from a single theoretical perspective, did not emerge as significant predictors in the cross-theoretical model. These included perceived threat and punishment certainty (rational choice theories), social norms (social theories), non-normative obligation (legitimacy theories), and impulsivity (capacity theories). Furthermore, authority evaluation and procedural justice (legitimacy theories) did not emerge as significant predictors in line with the results of the preceding analyses.

In sum, when looked at from a cross-theoretical perspective, only a limited subset of variables—drawn from legitimacy, capacity, and opportunity theories—emerged as significant predictors of compliance. All other predictors were reduced to nonsignificance.

## Discussion

The present study sought to develop a cross-theoretical approach to compli- ance that bridges and integrates the major theoretical approaches in the field of compliance studies. It proposed that to understand compliance in a particular setting, core variables from each of these theoretical strands should be considered simultaneously and systematically. It developed this approach in context of the massive, rule-induced behavioral changes that occurred dur- ing the first wave of the COVID-19 pandemic in Israel. We sought to demon- strate how a cross-theoretical approach to compliance in this setting can contribute beyond traditional, narrow approaches based on single theoreti- cal perspectives. From this, we sought to develop implications for our general understanding of compliance and the way that this might be studied in other settings.

### Pandemic Compliance During the First Wave in Israel

Our cross-theoretical approach revealed that the variables that ultimately explained compliance with mitigation measures at the time of our study origi- nated from a mix of distinct theoretical approaches, including legitimacy theories (moral alignment and general obligation to obey the law), capacity theories (capacity to comply), and opportunity theories (opportunity to violate). In this way, compliance in this setting was not just explained by individual motivational variables, such as agreement with the substance of the rules, but also by factors that relate to people’s practical circumstances and their physical environment. This observation is noteworthy in that much of the existing research on pandemic compliance during this period has focused on individual and social factors (e.g., Noone et al., 2021; Van Bavel et al., 2020). Conversely, environmental factors, which are prominent in criminological research (Clarke, 2003; Spano & Freilich, 2009), are not typically considered, except in research that focuses exclusively on macro-level processes (Brauner et al., 2021; Walsh et al., 2021).^31^ In sum, a cross-theoretical perspective underlines that to understand compliance in this setting, factors at each of these levels must be considered. Our findings revealed that when doing so, variables relating to legitimacy, capacity, and opportunity were important for explaining the massive behavioral changes that occurred during this period.

Equally noteworthy is the observation that when looked at from a cross- theoretical perspective, neither rational choice theories (costs and benefits, punishment) nor social theories (social norms) predicted compliance in the present empirical context; nor did other variables relating to legitimacy (e.g., procedural justice) or capacity (e.g., impulsivity). This could suggest that calculative, normative, and legitimacy-related factors were less influential due to the specific features of this setting—because, for example, compliance with mitigation measures was so widespread (i.e., low variability in norms), the situation so pressing (i.e., effectiveness may trump fairness considerations), or removal of opportunities for offending (e.g., closures of public places like schools or businesses, see Brauner et al., 2021; Walsh et al., 2021) rendered such considerations irrelevant.

### Implications of a Cross-Theoretical Approach

More relevant than the conclusions about what shapes compliance in this particular setting and time are the theoretical implications of the present results, the most important of which concerns the contrast between the results of the cross-theoretical regression model and the models informed by a single theoretical perspective. When looked at from a single theoretical perspective, in which key variables from all major strands of the compliance literature were tested in separate models, a wide range of variables emerged as significant predictors of compliance, originating from all five major theoretical families. However, when looked at from a cross theoretical perspective, in which key variables from the different theoretical approaches were simultaneously considered, many of these variables (perceived threat, punishment certainty, social norms, non-normative obligation, and impulsivity) no longer showed significant associations with compliance. Indeed, some theoretical approaches, specifically rational choice theories and social theories, became altogether irrelevant for explaining compliance. This result demonstrates that studies based on narrow theoretical perspectives may yield conclusions about compliance that no longer hold when a broader range of influences is considered. This also highlights an important risk for compliance practice: practical interventions that are based on such narrow perspectives may fail to be effective in practice. Of course, the question of what shapes compliance will differ between settings, and our conclusions on which factors matter (or do not matter) here should not be generalized directly; indeed, this is not our objective. Rather, the major implications of the present research are theoretical and methodological: to understand compliance requires a cross-theoretical perspective in which core mechanisms from across the major strands of the compliance literature are considered.

Of course, our research is not the first to combine different theoretical strands of the compliance literature. Indeed, compliance research often incor- porates selected variables from multiple theoretical perspectives. What the literature urgently lacks, however, is systematicity. Most studies include only a limited subset of variables, drawn from one, two, or three theories (e.g., see Gneezy & Rustichini, 2000; Murphy, 2003; Thornton et al., 2005; Wenzel, 2005). In this way, these approaches have omitted other major theories and variables that may be relevant to understanding compliance in their focal settings. This is even the case for the studies that have combined a greater number of theoretical strands and variables (e.g., Nielsen & Parker, 2012; Winter & May, 2001; Yan et al., 2016): these approaches have overlooked opportunity theories and only partially include capacity theories. The present findings underline that variables from each of these perspectives can be relevant for understanding how compliance is shaped; failure to include them may lead to misleading conclusions. A systematic approach like that here encompasses all major relevant theories, identifying key variables relevant to the setting, and considering each in the analysis. this can be contrasted to pragmatically focusing on a narrow subset of variables that reflect only some of the major theories. The latter studies will be limited in their practical ability to capture or incorporate all relevant influences. Nonetheless, our approach should not be seen as a recommendation to include every possible variable that research has found to be related to compliance. Rather, a systematic approach suggests that research should consider the major theoretical facets of what shapes compliance and use the key elements as they apply to the setting to better understand how compliance is shaped.

The contribution of our cross-theoretical approach does not lie only in its ability to illuminate compliance in particular situations. Rather, adopting a cross-theoretical perspective in different settings (for different rules, in dif- ferent jurisdictions, for different actors, in different regulatory settings) may also enable important theoretical advances for the general literature on com- pliance. Due to the patchworked, siloed nature of this scholarship, in which different literatures focus on different variables and do so across different settings, it lacks a clear understanding of how (and why) the processes that shape compliance may vary across different behaviors and settings. Studying compliance by means of a cross-theoretical approach can provide crucial insight. By systematically examining how key variables from across the main theories relate to compliance in different settings, compliance research can begin to map how core mechanisms rise or wane in importance in particular settings and begin to identify possible modulating factors. Furthermore, a cross-theoretical approach also may help to reconcile important inconsistencies in the compliance literature, One example is the deterrent effect of punishment, which have been demonstrated for traffic violations (Freeman et al., 2015; Hansen, 2015; Tay, 2005) but not for many other types of offenses (Nagin, 2013). For instance, in the present study, despite strong sanctions for violating pandemic mitigation measures in Israel or the beneficial effects of procedural fairness (which some regard as instrumental for compliance [e.g., Tyler et al., 2007], non-compliance still was present. In these ways, the cross-theoretical approach that we develop here may contribute to a more systematic understanding of compliance that shows what aspects of which theories best explain which rules, for which individuals and groups, and under what legal, socio-economic, and political conditions particular aspects of human nature and behavior are likely to be at play in how people respond to governmental rules. Moreover, this seems likely to produce a body of work that is directly relevant to practice by demonstrating which approaches, applied in what settings are likely to be effective,

One important avenue for future research is to understand more deeply the ways in which the different mechanisms in our cross-theoretical perspective interrelate. Given that many of the variables showed statistically significant associations with compliance when looked at from a single theoretical perspective, it is plausible that some of these variables have overlapping effects or may shape compliance indirectly, through their associations with more direct predictors. In the setting of pandemic compliance, for example, the compliance of others may not only signal social norms but may also enhance or constrain people’s capacity for compliance, as demonstrated by research on crowding (Hoeben et al., 2021; Liebst et al., 2022). Similarly, observed norms may signal how legitimate the measures and authorities seen to be. It is likely that different compliance mechanisms will interrelate in complex ways that go beyond the present research. By systematically mapping the core variables from different theoretical strands of the compliance literature, the cross-theoretical approach that we have developed here can also be used to explore such questions and further advance our understanding of how the different theories and variables operate to shape compliance (see Kuiper et al., 2022). These are important questions that future research may well explore.

### Practical Implications

What lessons can a cross-theoretical analysis of behavioral changes during the first pandemic wave teach for compliance practice? Care should be taking in generalizing these findings, as the processes that shape compliance are likely to differ between empirical settings, even for later stages of the COVID-19 pandemic due to changes in, for example, infections and, mitigation policies). Our approach, however, suggests some general lessons that may be valuable for compliance practice.

First and foremost, the cross-theoretical analysis that we have presented here highlights the possible risks of developing practical interventions on the basis of narrow approaches to compliance. As the present findings demon- strate, variables that predict compliance in studies that include only a limited subset of variables and theories may yield misleading conclusions. Accordingly, leveraging such conclusions in practical interventions may be ineffective or possibly even produce detrimental effects. In the present setting, for example, a narrow approach might have identified punishment as a viable target for practical intervention. However, when all key compliance theories and variables were considered, its impact on compliance was negligible. Indeed, increasing punishment would have done nothing to leverage the most influential predictor in the cross-theoretical analysis: capacity. Our findings, then, suggest that practical interventions to promote compliance are best based on broad approaches to compliance, in which key mechanisms are assessed systematically, such as with the cross-theoretical approach that we have presented here.

Second, our findings may also serve as an important reminder of the importance of considering people’s practical capacity for complying and their opportunities for offending. Practical interventions frequently target incentives, motives, and norms (e.g., sanctioning systems, codes of conduct), but they have tended to devote less attention to the question of whether people are actually capable of doing what such measures require of them or what environmental barriers may exist (Gray & Silbey, 2011). Indeed, if people do not know what the rules demand of them, face conflicting demands, or confront practical obstacles that make it impossible for them to respond appropriately, compliance is unlikely, no matter the sanctions, pledges, or perceived norms. The present findings may serve as an important reminder of the role that such practical factors play in shaping compliance and of how these could be leveraged by interventions that enhance people’s capacity to comply (e.g., knowledge, practical resources), or shape their environments to facilitate their doing so.

### Limitations

The present findings are situated in the specific empirical context of this study. We cannot directly generalize these findings outside of this context. Accordingly, our results do not allow us to directly explain compliance with COVID-19 mitigation measures in other countries, or even in Israel following the first pandemic wave. This applies even more for attempts to generalize our findings to compliance with other rules or policies. It should be noted, however, that this was not the purpose of our study. Rather, it is the cross-theoretical approach that we have presented here that should be generalized to other settings, to understand how the core theories and variables that we have identified here may shape compliance there.

Furthermore, we should note some limitations to our measures. First, the measures rely on self-reports, which may be vulnerable to imperfect recall or social desirability bias (Bauhoff, 2011; Van de Mortel, 2008). The observed high levels of self-reported compliance do align, however, with objective measures of compliance during this period.^32^ Moreover, our approach enabled us to provide insight that is impossible to obtain with more aggregated, distal data: namely, into the individual-level variables that explain variation in (self-reported) compliance. Self-report measures have been used extensively in research on pandemic mitigation measures, and some studies have shown only a limited impact of social desirability (Larsen et al., 2020).

Finally, our theoretical focus meant that we did not aim to evaluate the policy response in Israel or to zero in on the country’s particular social, religious, and political dynamics (Barak Corren & Perry-Hazan, 2021; Gesser-Edelsburg et al., 2020; Waitzberg et al., 2020). As such, we did not set out to collect a sample that was fully representative of the Israeli population. To understand how the processes that shape compliance may operate in the broader Israeli population, in specific ethnic and religious communities, or in the period beyond the critical first pandemic wave, further research is needed that focuses directly on these questions.

### Conclusion

To understand why people obey or break rules, different approaches have formulated different theories, and have zoomed in on different subsets of variables. In the setting of the massive behavioral changes of the first wave of the COVID-19 pandemic in Israel, the present research developed a cross-theoretical approach in which these perspectives are integrated. Our analysis revealed that a cross-theoretical perspective can importantly deepen our understanding of why people comply by revealing how compliance is shaped through the interplay of rational calculation, social influence, perceptions of legitimacy, practical capacity, and situational opportunities.

## Supplemental Material

sj-docx-1-aas-10.1177_00953997221140899 – Supplemental material for Cross-Theoretical Compliance: An Integrative Compliance Analysis of COVID-19 Mitigation Responses in Israel Click here for additional data file. Supplemental material, sj-docx-1-aas-10.1177_00953997221140899 for Cross-Theoretical Compliance: An Integrative Compliance Analysis of COVID-19 Mitigation Responses in Israel by Anne Leonore de Bruijn, Yuval Feldman, Christopher P. Reinders Folmer, Malouke E. Kuiper, Megan Brownlee, Emmeke Kooistra, Elke Olthuis, Adam Fine and Benjamin van Rooij in Administration & Society

## Notes

1.
https://www.jpost.com/Breaking-News/529-Israelis-have-been-diagnosed-with-coronavirus-Health-Ministry-621536

2. https://www.calcalist.co.il/local/articles/0,7340,L-3807777,00.html (Hebrew)

3.
https://hamadad-haleumi.co.il/

4.
https://www.al-monitor.com/pulse/originals/2020/04/israel-idf-soldiers-police-coronavirus-ultra-orthodox.html

5.
https://www.covid19healthsystem.org/_layouts/livinghit/customforms/dispnews.aspx?id=33&site= https://www.covid19healthsystem.org/countries/israel&isdlg=1

6.
https://www.jpost.com/Israel-News/Coronavirus-cases-climb-to-77-second-case-of-unknown-origin-confirmed-620578

7.
https://www.jpost.com/Breaking-News/529-Israelis-have-been-diagnosed-with-coronavirus-Health-Ministry-621536

8.
https://www.gov.il/en/departments/news/25032020_01

9.
https://covidtracker.bsg.ox.ac.uk/stringency-scatter

10.
https://www.gov.il/en/departments/news/25032020_01

11.
https://www.haaretz.com/israel-news/coronavirus-cases-in-israel-spike-to-164-nearly-2-500-medical-officials-quarantined-1.8671075

12.
https://www.nytimes.com/2020/04/12/world/middleeast/coronavirus-israel-mossad.html

13.
https://home.kpmg/xx/en/home/insights/2020/04/israel-government-and-institu-tion-measures-in-response-to-covid.html

14. https://news.walla.co.il/item/3348795 (Hebrew)

15.
https://www.zman.co.il/91518/

16.
https://hamadad-haleumi.co.il/

17.
https://www.al-monitor.com/pulse/originals/2020/04/israel-idf-soldiers-police-coronavirus-ultra-orthodox.html

18.
https://www.jpost.com/Breaking-News/Intern-at-Ichilov-contracts-coronavirus-as-Israeli-cases-spike-to-250-621142

19. https://www.timesofisrael.com/israel-bars-gatherings-over-2000-people-limits-visits-to-sick-or-elderly/ and https://www.jpost.com/Israel-News/Coronavirus-cases-climb-to-77-second-case-of-unknown-origin-confirmed-620578

20.
https://www.jpost.com/HEALTH-SCIENCE/Coronavirus-Teachers-Union-calls-on-Education-Ministry-to-shutter-schools-620688

21.
https://www.timesofisrael.com/jerusalem-ultra-orthodox-neighborhoods-to-be-locked-down-starting-sunday/

22.
https://www.timesofisrael.com/cabinet-declares-bnei-brak-restricted-zone-read-ies-to-do-same-for-other-towns/

23.
https://govextra.gov.il/ministry-of-health/corona/corona-virus-en/guidelines/

24.
https://www.gov.il/en/Departments/news/25032020_01

25. Panel4All awards points for participating that can eventually be traded for money. The points awarded for this study represented a value of approximately six NIS.

26. The attention check item stated: This question is to verify you are reading the questions, please respond “Disagree.”

27. This variable was only recorded among participants who indicated Jewish for ethnicity. Because only small subsamples were obtained for Orthodox (*N* = 36) and Ultra-orthodox (*N* = 25), this variable was treated as an ordinal measure, with higher scores indicating stricter religiosity.

28. Sixty-four participants preferred to not disclose their religion, and 74 partici- pants chose to not reveal their political orientation. These participants were omit- ted in analyses that included these variables.

29. Similar to our studies in the United States (Van Rooij et al., 2020), the United Kingdom (Kooistra et al., 2020), and the Netherlands (Kuiper et al., 2022), our survey also included a measure assessing people’s knowledge of the mitigation measures. However, contrary to these other studies, our survey in Israel measured knowledge using Likert scales (i.e., the extent to which people think that these measures apply or not), instead of binary measures (whether the measures apply or not). In hindsight, the Likert format is poorly suited to assessing knowledge, as it lacks a clear reference standard that can be used to identify responses as true or false (i.e., it is not clear whether greater agreement indicates greater correctness, as opposed to true/false measures which can factually be compared to the rules that apply). For this reason, we did not include these items in our analyses.

30. Because religion (secular, traditional, orthodox, or ultra-orthodox) was only measured among participants who selected Israeli as their ethnicity (and not among those who selected Arab), a correlation between these variables could not be computed.

31. Although Noone et al. (2021) suggest in a recent scoping review that most stud- ies on social distancing have examined “Environmental Context and Resources,” a closer examination of their data reveals that this category chiefly consists of individual demographic variables (e.g., age, gender, education, etc.; subcategory “Person × environment interaction”) and other governmental mitigation policies (e.g., shelter-in-place orders, non-essential business closure, etc., subcategory “Barriers and facilitators”), and rarely refers to features of people’s physical environment.

32.
https://www.gstatic.com/covid19/mobility/2020-04-05_IL_Mobility_Report_en.pdf
