# Supplementary material for: Cross-Theoretical Compliance: An Integrative Compliance Analysis of COVID-19 Mitigation Responses in Israel
Source: Adm Soc. 2022 Dec 22;55(4):635–70. doi: 10.1177/00953997221140899 (PMC9790859; doi:10.1177/00953997221140899)
Supplement: sj-docx-1-aas-10.1177_00953997221140899 – Supplemental material for Cross-Theoretical Compliance: An Integrative Compliance Analysis of COVID-19 Mitigation Responses in Israel [file sj-docx-1-aas-10.1177_00953997221140899.docx]

**Appendix**

**Survey Questionnaire Items**

**Demographic and control variables**

The following demographic and control variables were recorded: Age, gender, ethnicity, religion (secular, traditional, orthodox, or ultra-orthodox), education, number of people in household, number of children, and social economic status before and after COVID-19 (based on the MacArthur Scale of Subjective Social Status; (Adler et al., 2000)). Furthermore, we measured whether participants provided professional care for COVID-19 patients, had friends or family over the age of 75, and whether they or anyone they knew had underlying health issues that placed them at increased risk of COVID-19. Last, we measured participants’ trust in science and trust in media (adapted from McCright et al. (2013)): “Please indicate how much you trust scientists to create knowledge that is unbiased and accurate,” and “Please indicate how much you trust traditional media (e.g., newspapers, TV news, news apps) reporting on the Coronavirus to be unbiased and accurate” (1 = “completely distrust,” 5 = “completely trust”). Political orientation was measured with a single item adapted from Fine et al. (2019): “How would you describe your political view?” (1 = “very progressive,” 4 = “very conservative”).

**Compliance**

Four items measured whether participants complied with social distancing measures: “I still meet people outside of my direct household,” “I keep a safe distance from people outside of my direct household,” “I still visit others (friends, relatives) outside of my direct household,” and “I still allow others (friends, relatives) to visit my direct household” (1 = “never,” 7 = “always”). Items 1, 3, and 4 were reverse coded. One item measured whether participants complied with stay-at-home measures: “I have stayed at home after I was ordered to do so, apart from engaging in essential activities (e.g., grocery shopping, medical appointments).” For all five (recoded) items, higher scores indicated greater compliance.

Our compliance measures did not display sufficient internal consistency to be combined into a scale measure (α = .64). For this reason, we decided instead to analyze not the *degree* of compliance (as captured by the Likert measures), but rather the *frequency* of (full) compliance (please see the main text for more details).

**Rational choice theories**

Four variables rooted in rational choice approaches to compliance were assessed: (1) perceived costs of compliance, (2) threat perceptions, (3) perceived punishment certainty, and (4) perceived punishment severity.

**Costs of compliance.** Participants indicated on five items (α = .77) how likely it was that the mitigation measures would have a negative impact on their life or livelihood: (Due to the measures to contain the Coronavirus, I will likely…) “…lose income,” “…lose my job,” “…not be able to work,” “…not be able to work as effectively as normal,” and “…experience a negative impact on my social life” (1 = “extremely unlikely,” 7 = “extremely likely”). Mean scores were calculated, with higher values indicating greater perceived costs of compliance.

**Perceived threat.** Perceived threat was measured using two items, where participants indicated to what extent they believed the Coronavirus was a threat to their own health or that of the general public: (I believe the Coronavirus is a major threat…) “…to my own health,” and “…to the general health” (1 = “very strongly disagree,” 7 = “very strongly agree”). The two items were closely associated (*r* = .56, *p* < .001), and therefore were averaged into a single scale measure, with higher scores indicating greater perceived threat.

**Punishment certainty.** Two items measured perceived certainty of apprehension and punishment for violating social distancing measures: (How probable is it that authorities will…) “…find out if you do not follow social distancing measures?” and “…punish you if you do not follow social distancing measures?” (1 = “extremely improbable,” 7 = “extremely probable”). Two further items asked the same for lockdown measures. Both sets of measures were highly correlated (*r*s ≥ .51, *p* < .001), and hence were averaged into a single scale measure (α = .87), with higher scores indicating greater perceived certainty of punishment.

**Punishment severity.** One item assessed participants’ expectations of the severity of punishment for violating social distancing measures: “How much will you suffer if authorities punish you not following social distancing measures?” (1 = “extreme suffering,” 6 = “no suffering at all”). Another question asked the same for lockdown measures. Both items were highly correlated (*r* = .88, *p* < .001) and thus were mean-scored into a single scale measure. Higher scores indicated greater perceived severity of punishment.

**Social theories**

One variable rooted in social approaches to compliance was measured: perceived descriptive social norms. Participants rated to what extent most people they knew “are following social distancing measures,” and “are following lockdown measures” (1 = “very strongly disagree,” 7 = “very strongly agree”). Responses on both items were highly correlated (*r* = .94, *p* < .001) and were therefore averaged into a scale measure, with higher scores indicating more compliant perceived social norms.

**Legitimacy theories**

Four variables reflecting legitimacy and procedural justice were assessed: (1) moral alignment, (2) authority evaluation, (3) obligation to obey, and (4) procedural justice.

**Moral alignment.** Moral alignment was measured using two items, where participants indicated to what extent they believed people should follow the COVID-19 mitigation measures: (I morally believe people should…) “…follow social distancing measures to contain the Coronavirus,” and “…follow stay at home measures to contain the Coronavirus” (1 = “very strongly disagree,” 7 = “very strongly agree”). The two items were closely associated (*r* = .86, *p* < .001), and therefore were mean-scored into a single scale measure. Higher scores indicated greater moral alignment.

**Authority evaluation.** Evaluations of the authority response to the pandemic were measured using three items: (Authorities and government officials…) “…have been consistent with their approach to containing the Coronavirus,” “…have responded adequately with their approach to containing the Coronavirus,” and “have gone too far with their approach to containing the Coronavirus” (1 = “very strongly disagree,” 7 = “very strongly agree”). Item 3 showed a poor item-total correlation, however, and the internal consistency of the scale was greatly improved when it was omitted (form α = .71 to α = .86). As such, items 1 and 2 were averaged into a scale measure, with higher scores indicating more favorable authority evaluations.

**Obligation to obey.** Our survey assessed two facets of people’s obligation to obey: (a) their obligation to obey legal rules in general (Estévez & Emler, 2010; Fine et al., 2016; Fine & Van Rooij, 2021), and (b) their obligation to obey authorities out of a sense of coercion or fear (i.e., non-normative obligation to obey, see Posch et al. (2020)). General obligation to obey the law (general OOL) was measured with a single item, based on Estévez and Emler (2010): “I feel like it is sometimes okay to break the law” (1 = “strongly agree,” 7 = “strongly disagree”). Higher scores indicated greater general obligation to obey the law. Non-normative obligation to obey was measured using a single item (adapted from Posch et al. (2020); Tankebe et al. (2016)): “I only obey the authorities handling the Coronavirus because I am afraid of them” (1 = “very strongly disagree,” 7 = “very strongly agree”). Higher scores indicated greater non-normative obligation to obey.

**Procedural justice.** Procedural justice (PJ) was measured using items based on prior research on fairness perceptions and law (Baker & Gau, 2018; Gau, 2014; Tyler, 1997; Wolfe et al., 2016). Following the conceptualization of Tyler (2006), we distinguished both the perceived fairness of decision-making procedures and that of one’s treatment by decision-makers. As such, three items (α = .93) measured perceived procedural justice in the creation of the mitigation measures: (In creating measures to contain the Coronavirus, I expect that government officials…) “…will treat people with respect,” “will treat people fairly, despite gender, race, religion, or socio-economic background,” and “will be honest in creating measures to contain the Coronavirus” (1 = “very strongly disagree,” 7 = “very strongly agree”). Four items (α = .92) measured perceived procedural justice in their enforcement: (In enforcing the measures to contain the Coronavirus, I expect that government officials…) “…will treat people with respect,” “…will give a person the chance to tell their side of the story if the person is accused of violating measures to contain the Coronavirus,” “…will treat people fairly, despite gender, race, religion, or socio-economic background,” and “…will be honest in enforcing measures to contain the Coronavirus” (1 = “very strongly disagree,” 7 = “very strongly agree”). The resulting two scale measures were highly correlated (*r* = .83, *p* < .001). Therefore, a combined scale was constructed by averaging responses on all seven items (α = .95), with higher values indicating higher perceived procedural justice.

**Capacity theories**

Three variables derived from capacity approaches to compliance were measured: (1) capacity to comply, (2) impulsivity, and (3) negative emotions.^[[1]](#endnote-1)^

**Capacity to comply.** Three items measured to what extent participants were practically able to comply with the COVID-19 mitigation measures: “At this moment, I am able to keep a safe distance from others,” “At this moment, I work from home as much as possible,” and “At this moment, I am able to not go outside for anything but essential activities (e.g., grocery shopping, medical appointments)” (1 = “very strongly disagree,” 7 = “very strongly agree”). Item 2 correlated poorly with the other two items, and internal consistency was greatly improved after its omission (form α = .62 to α = .81). The two remaining items were averaged into a scale measure, with higher scores indicating greater capacity to comply.

**Impulsivity.** Impulsivity was measured using a subset of five items (α = .75) taken from the 8-item impulse control subscale from the Weinberger Adjustment Inventory (WAI, Weinberger and Schwartz (1990)): “I should try harder to control myself when I’m having fun,” “I do things without giving them enough thought,” “When I’m doing something fun (like partying or acting silly), I tend to get carried away and go too far,” “I say the first thing that comes to my mind without thinking enough about it,” and “I stop and think things through before I act” (1 = “false,” 5 = “true;” item 5 was reverse coded). Responses were mean-scored into a scale measure; higher scores indicated greater impulsivity.

**Negative emotions.** Negative emotions due to COVID-19 were measured using six items (α = .87): (The Coronavirus makes me feel…) “…angry,” “…anxious,” “powerless,” “…depressed,” “…stressed,” and “lonely” (1 = “very strongly disagree,” 7 = “very strongly agree”). Responses were averaged into a scale measure and higher values indicated more negative emotions.

**Opportunity theories**

One variable rooted in opportunity approaches to compliance was assessed: opportunity to violate. Five items (α = .86) measured participants’ perceived opportunities for violating the mitigation measures: (At this moment, if it were against the rules, it would still be possible for me…) “…to be in close proximity to people outside my direct household,” “…to go to work,” “to meet people outside of my direct household,” “to go outside for non-essential activities,” and “to go to public venues where many people gather” (1 = “very strongly disagree,” 7 = “very strongly agree”). Responses were mean scored into a scale measure, with higher values indicating greater perceived opportunities for violating.

**References**

Adler, N. E., Epel, E. S., Castellazzo, G., & Ickovics, J. R. (2000). Relationship of subjective and objective social status with psychological and physiological functioning: Preliminary data in healthy white women. *Health Psychology*, *19*(6), 586-592. <https://doi.org/10.1037/0278-6133.19.6.586>

Baker, T., & Gau, J. M. (2018). Female offenders’ perceptions of police procedural justice and their obligation to obey the law. *Crime & Delinquency*, *64*(6), 758-781. <https://doi.org/10.1177/0011128717719418>

Estévez, E., & Emler, N. P. (2010). A structural modelling approach to predict adolescent offending behaviour from family, school and community factors. *European Journal on Criminal Policy and Research*, *16*(4), 207-220.

Fine, A., Van Rooij, B., Feldman, Y., Shalvi, S., Leib, M., Scheper, E., & Cauffman, E. (2016). Rule Orientation and behavior: Development and validation of a scale measuring individual acceptance of rule violation. *Psychology, Public Policy, and Law 22*(3), 314-329. <https://doi.org/10.1037/law0000096>

Fine, A. D., Rowan, Z., & Simmons, C. (2019). Do politics trump race in determining America's youths' perceptions of law enforcement? *Journal of Criminal Justice*, *61*, 48-57. <https://doi.org/10.1016/j.jcrimjus.2019.01.003>

Fine, A. D., & Van Rooij, B. (2021). Legal Socialization: Understanding the Obligation to Obey the Law. *Journal of Social Issues*, *77*(2), 367-391. <https://doi.org/10.1111/josi.12440>

Gau, J. M. (2014). Procedural justice and police legitimacy: A test of measurement and structure. *American Journal of Criminal Justice*, *39*(2), 187-205. <https://doi.org/10.1007/s12103-013-9220-8>

McCright, A. M., Dentzman, K., Charters, M., & Dietz, T. (2013). The influence of political ideology on trust in science. *Environmental Research Letters*, *8*(4), 044029. <https://doi.org/10.1088/1748-9326/8/4/044029/meta>

Posch, K., Jackson, J., Bradford, B., & Macqueen, S. (2020). ” Truly Free Consent”? Clarifying the Nature of Police Legitimacy using Causal Mediation Analysis. *Journal of Experimental Criminology*.

Tankebe, J., Reisig, M. D., & Wang, X. (2016). A multidimensional model of police legitimacy: A cross-cultural assessment. *Law and Human Behavior*, *40*(1), 11.

Tyler, T. R. (1997). Procedural Fairness and Compliance with the Law. *Swiss Journal of Economics and Statistics*, *133*(2), p. 219-240.

Tyler, T. R. (2006). *Why People Obey the Law*. Princeton University Press.

Weinberger, D. A., & Schwartz, G. E. (1990). Distress and restraint as superordinate dimensions of self‐reported adjustment: A typological perspective. *Journal of Personality*, *58*(2), 381-417. <https://doi.org/10.1111/j.1467-6494.1990.tb00235.x>

Wolfe, S. E., Nix, J., Kaminski, R., & Rojek, J. (2016). Is the effect of procedural justice on police legitimacy invariant? Testing the generality of procedural justice and competing antecedents of legitimacy. *Journal of Quantitative Criminology*, *32*(2), 253-282. <https://doi.org/10.1007/s10940-015-9263-8>

1. Similar to our studies in the United States (Authors 2020c), the United Kingdom (Authors 2020a), and the Netherlands (Authors 2020b), our survey also included a measure assessing people’s knowledge of the mitigation measures. However, contrary to these other studies, our survey in Israel measured knowledge using Likert scales (i.e., the extent to which people think that these measures apply or not), instead of binary measures (whether the measures apply or not). In hindsight, the Likert format is poorly suited to assessing knowledge, as it lacks a clear reference standard that can be used to identify responses as true or false (i.e., it is not clear whether greater agreement indicates greater correctness, as opposed to true/false measures which can factually be compared to the rules that apply). For this reason, we did not include these items in our analyses. [↑](#endnote-ref-1)
